# Hybrid Carbon Supports Composed of Small Reduced Graphene Oxide and Carbon Nanotubes for Durable Oxygen Reduction Catalysts in Proton Exchange Membrane Fuel Cells

**DOI:** 10.3390/ijms232113312

**Published:** 2022-11-01

**Authors:** Su-Jeong Bak, Mingyu Son, Jeehoon Shin, Sun-I Kim, Jung Woo Lee, Duck Hyun Lee

**Affiliations:** 1 Green Materials and Processes R&D Group, Korea Institute of Industrial Technology, Ulsan 44413, Korea; 2 Department of Materials Science & Engineering, Pusan National University, Busan 46241, Korea

**Keywords:** oxygen reduction reaction, proton exchange membrane fuel cell, graphene, carbon nanotube, Pt catalyst

## Abstract

We demonstrated highly active and durable hybrid catalysts (HCs) composed of small reduced graphene oxide (srGO) and carbon nanotubes (CNTs) for use as oxygen reduction reaction (ORR) catalysts in proton exchange membrane fuel cells. Pt/srGO and Pt/CNTs were prepared by loading Pt nanoparticles onto srGO and CNTs using a polyol process, and HCs with different Pt/CNT and Pt/srGO ratios were prepared by mechanically mixing the two components. The prepared HCs consisted of Pt/CNTs well dispersed on Pt/srGO, with catalyst HC55, which was prepared using Pt/srGO and Pt/CNTs in a 5:5 ratio, exhibiting excellent oxygen reduction performance and high stability over 1000 cycles of the accelerated durability test (ADT). In particular, after 1000 cycles of the ADT, the normalized electrochemically active surface area of Pt/HC55 decreased by 11.9%, while those of Pt/srGO and Pt/C decreased by 21.2% and 57.6%, respectively. CNTs have strong corrosion resistance because there are fewer defect sites on the surface, and the addition of CNTs in rGO further improved the durability and the electrical conductivity of the catalyst. A detailed analysis of the structural and electrochemical properties of the synthesized catalysts suggested that the synergetic effects of the high specific surface area of srGO and the excellent electrical conductivity of CNTs were responsible for the enhanced efficiency and durability of the catalysts.

## 1. Introduction

Proton exchange membrane fuel cells (PEMFCs) are considered clean energy sources for both stationary systems and portable devices because of their low operating temperature, fast startup, high energy efficiency, and high power density [1,2,3]. Since most automotives operate for 5000–20,000 h, the long-term durability of PEMFCs is essential for their commercial use. In particular, durable oxygen reduction reaction (ORR) catalysts that can endure long operation times are essential because the ORR at the cathode is the rate-limiting reaction step in PEMFCs. Pt nanoparticles supported on carbon materials with a high specific surface area, such as carbon black (CB), are widely used as catalysts in PEMFCs. However, CB is thermodynamically unstable under the operating conditions of PEMFCs, and the carbon oxidation reaction (COR) is accelerated at elevated temperatures and potentials. The COR causes the detachment or agglomeration of Pt nanoparticles owing to the weakening of the interactions between the nanoparticles and the carbon support, resulting in a decrease in the Pt surface area [3,4].

To improve the long-term durability of ORR catalysts, carbon nanomaterials such as carbon nanotubes (CNTs), graphene, and reduced graphene oxide (rGO) have been explored for use as support materials for Pt nanoparticles [5,6,7]. CNTs exhibit numerous desirable properties, including a large specific surface area, excellent electrochemical durability, high electrical conductivity, and excellent mechanical strength. When CNTs are used as the support for ORR catalysts, the high crystallinity of the CNTs enhances the conductivity of the catalysts, while the high specific surface area and large number of mesopores in the CNTs result in the high dispersibility of the Pt nanoparticles [3,5,6,8,9,10,11,12,13]. Wang et al. demonstrated that a CNT support can suppress the sintering of Pt nanoparticles. In addition, several studies have reported a reduction in Pt usage and improvements in fuel cell performance with the use of a CNT-supported Pt catalyst. Graphene has also attracted significant attention as a durable catalyst support owing to its high conductivity and large specific surface area [14,15,16,17]. We had recently reported highly efficient ORR catalysts composed of uniform Pt nanoparticles supported on small rGO (srGO). The performance and stability of these Pt/srGO catalysts were better than those of Pt/rGO and Pt/CB catalysts [3].

In this study, we synthesized highly durable hybrid catalysts (HC) composed of srGO and CNTs for use as ORR catalysts in PEMFCs. First, Pt/srGO and Pt/CNTs were prepared by loading Pt nanoparticles onto srGO and CNTs using a polyol process. Subsequently, HCs with different Pt/CNT and Pt/srGO ratios were prepared by mechanically mixing the two catalysts. The characteristics of the synthesized catalysts were analyzed using scanning electron microscopy (SEM), transmission electron microscopy (TEM), thermogravimetric analysis, and Raman spectroscopy. Their electrochemical characteristics were evaluated using linear sweep voltammetry (LSV) and cyclic voltammetry (CV), which were performed using single cells. The results confirmed that the HCs showed superior electrochemical properties compared with those of CB, CNTs, and srGO in terms of the ORR activity and long-term durability.

## 2. Results and Discussion

### 2.1. Morphology and Crystal Structure

**Figure 1:**
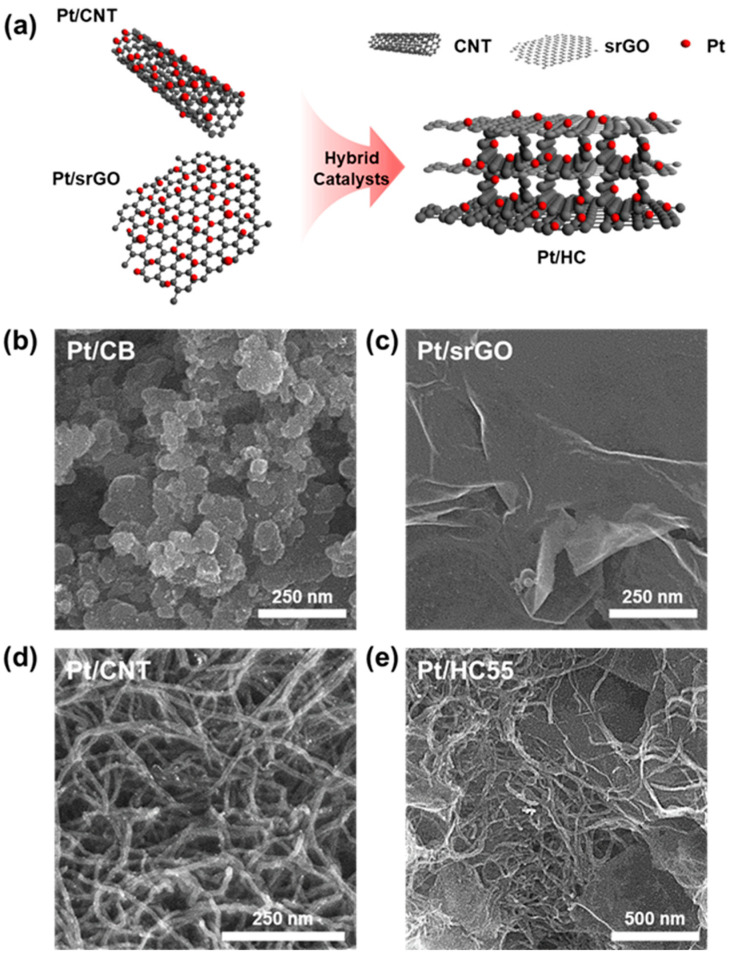
(**a**) Schematic of synthesis of ORR hybrid catalysts. SEM images of (**b**) Pt/CB, (**c**) Pt/srGO, (**d**) Pt/CNTs, and (**e**) Pt/HC55.

Figure 1a shows a schematic of the process for synthesizing the HCs. As stated previously, the HCs were prepared by the mechanical mixing of Pt/CNTs and Pt/srGO. To determine the optimal mixing ratio for Pt/srGO and Pt/CNTs, they were mixed in ratios of 7:3 (Pt/HC73), 5:5 (Pt/HC55), and 3:7 (Pt/HC37). The morphologies of Pt/C, Pt/srGO, Pt/CNTs, Pt/HC73, Pt/HC55, and Pt/HC37 were observed using SEM (Figure 1b–e and Figure S1). As the proportion of CNTs in the HCs was increased, more CNTs with a diameter of 20 nm were distributed on the surfaces of the srGO sheets, which had an average size of 2.6 μm.

**Figure 2:**
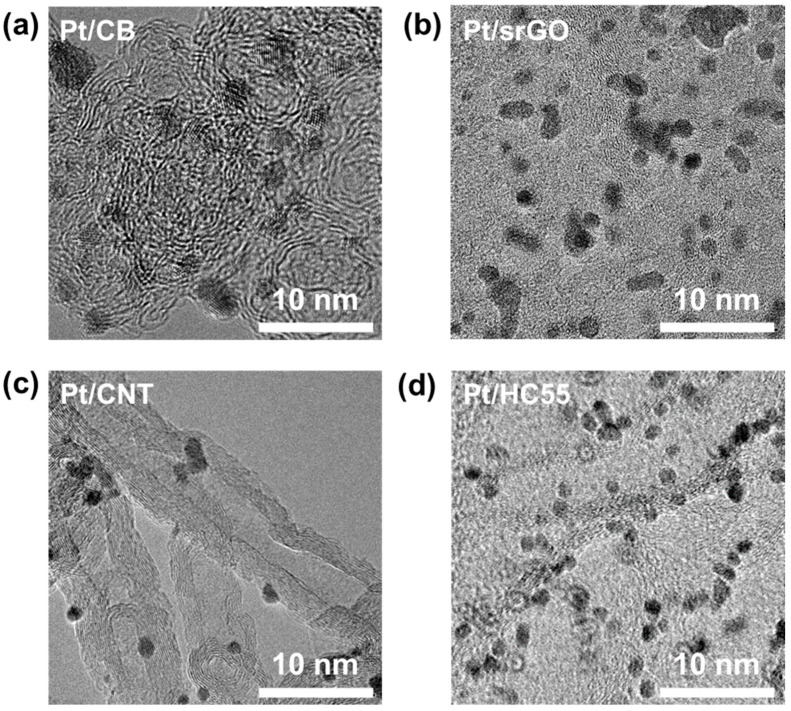
TEM images of (**a**) Pt/CB, (**b**) Pt/srGO, (**c**) Pt/CNTs, and (**d**) Pt/HC55.

The morphology of the Pt nanoparticles in the synthesized catalysts was observed using TEM (Figure 2). Uniform-sized Pt nanoparticles were well dispersed in all the catalysts, and their average size was 2–3 nm (Figure S2). XRD analysis was performed to determine the crystalline structures of the Pt nanoparticles on the carbon supports. The XRD patterns of Pt/srGO, Pt/HC73, Pt/HC55, Pt/HC37, and Pt/CNTs contained peaks related to the (111) and (200) planes of the face-centered cubic structure of crystalline Pt at 2θ = 40° and 46° (Figure S3a) [18,19]. The thermogravimetric analysis results confirmed that the content of Pt loading weight was the same, with 20 wt% in all the prepared catalysts (Figure S3b). Brunauer–Emmett–Teller (BET) analysis was performed to determine the specific surface areas and pore volumes of Pt/srGO, Pt/HC73, Pt/HC55, Pt/HC37, and Pt/CNTs (Table 1) [20,21,22].

**Figure 3:**
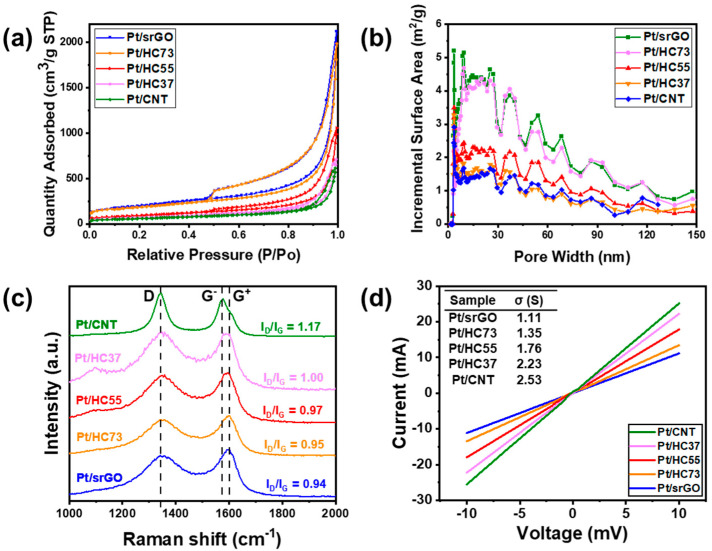
(**a**) BET nitrogen adsorption isotherm plot, (**b**) NL-DFT pore size distribution calculated from N_2_ adsorption isotherms, (**c**) Raman spectra, and (**d**) representative IV curves of Pt/srGO, Pt/CNT, Pt/HC73, Pt/HC55 and Pt/HC37.

The results confirmed that the specific surface area of Pt/srGO, which consisted of pure srGO as the support, was the largest because of the high specific surface area of srGO. The specific surface area gradually decreased as the amount of CNTs in the HCs increased. Figure 3a presents the N_2_ adsorption–desorption isotherms of all catalysts, and they can be classified as Type IV isotherms with a H1-type hysteresis loop. These isotherms are characteristic of the mesoporous nature of the synthesized materials. Figure 3b exhibits the NL–DFT pore size distribution calculated from the N_2_ adsorption isotherms of the prepared catalysts, and it also confirms that the prepared catalysts were mesoporous. The Raman spectra of the catalysts confirmed that the ratio of the D-band intensity (I_D_) to the G-band intensity (I_G_) (i.e., ID/IG) increased with the amount of CNTs used (Figure 3c) [23,24]. It can be said that, in the absence of a pretreatment, the surfaces of the CNTs were amorphous and showed low crystallinity and hence a high peak intensity ratio. Therefore, an increase in the proportion of CNTs increased the ID/IG ratio. Figure 3d shows the electrical characteristics—that is, the current (I)–voltage (V) characteristics—of the catalysts, as determined using a source measure unit. The electrical resistances of Pt/srGO, Pt/CNT, and the HCs were determined. The electrical resistance of Pt/srGO was the highest. Moreover, the electrical resistance of the HCs decreased as their CNT content was increased. Pt/CNTs exhibited the lowest resistance. This is because srGO has higher resistance than that of structurally stable CNTs, owing to the presence of a greater number of defects in the former because of its small size. Generally, rGO is synthesized by the oxidation of graphite in a potassium permanganate and concentrated sulfuric acid, followed by the reduction process with ascorbic acid. Although rGO is reduced, it still has many oxygen functional groups on the surface, which increases the electrical resistance of rGO. Furthermore, the number of dangling defects on rGO increases with the decrease in rGO size, and the electrical resistance is also increased because the contact resistance of bulk rGO increases [3]. Thus, the Pt/srGO showed relatively high electrical resistance. In the case of the HCs, Pt/HC73, which had high srGO content, showed the lowest conductivity, while Pt/HC37 showed the highest conductivity. Therefore, an increase in the CNT content of the HCs increased their electrical conductivity and decreased their specific surface area.

### 2.2. Electrocatalytic Performance 

**Figure 4:**
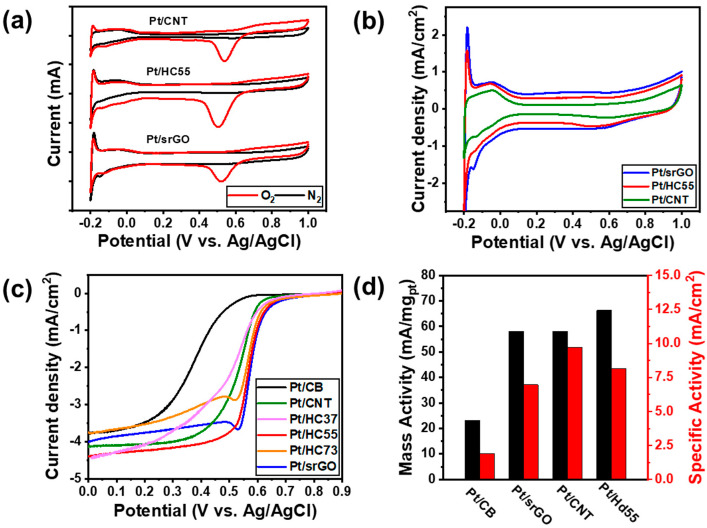
(**a**) CV curves of Pt/CB, Pt/CNT, Pt/srGO, Pt/HC37, Pt/HC55, and Pt/HC73 in N_2_-saturated (black lines) and O_2_-saturated (red lines) 0.5 M H_2_SO_4_ at a scan rate of 20 mV/s. (**b**) CV curves of the catalysts in N_2_-saturated 0.5 M H_2_SO_4_ at a scan rate of 20 mV/s. (**c**) Polarization curves of the catalysts in O_2_-saturated 0.5 M H_2_SO_4_ at a scan rate of 5 mV/s and GCE rotational speed of 1600 rpm. (**d**) Mass and specific activities of the catalysts at 0.4 V vs. Ag/AgCl.

To examine the fuel cell performance of the catalysts, we performed LSV and CV measurements. Figure 4a shows the typical CV curves of Pt/srGO, Pt/CNTs, and Pt/HC55. All the catalysts exhibited a large cathodic peak at 0.55 when an O_2_-saturated 0.5 M H_2_SO_4_ solution was used as the electrolyte; however, this peak was not observed when a N_2_-saturated electrolyte was used. With respect to the O_2_-saturated electrolyte, Pt/HC55 had a larger cathodic peak that those of the catalysts based on rGO or CNTs alone. Figure 4b shows the CV curves of Pt/srGO, Pt/CNTs, and the HCs; the current density in the double-layer region was the highest for Pt/srGO (because of the relatively large surface area of srGO) and the lowest for Pt/CNT. That of Pt/HC55, which contained srGO and CNTs in equal proportions, lay in between. Figure 4c shows the polarization curves of Pt/C, Pt/srGO, Pt/CNTs, Pt/HC73, Pt/HC55, and Pt/HC37, as measured at a rotation speed of 1600 rpm. Pt/HC55 exhibited a much higher onset potential of 614.3 mV (versus Ag/AgCl) than that of Pt/C (489.6 mV), and it was similar to that of Pt/srGO (612.6 mV) and Pt/CNTs (610.3 mV). Furthermore, the half-wave potential (E_half-wave_) of Pt/HC55 (580.2 mV) was larger than those of Pt/C (318.05 mV), Pt/srGO (568.8 mV), and Pt/CNTs (533.0 mV). Owing to the high electroconductivity of the CNTs and the high specific area of srGO, the electrochemical properties of Pt/HC55 were better than those of Pt/C, Pt/srGO, and Pt/CNTs. We determined the mass activity (MA) and specific activity (SA) at 0.4 V (versus Ag/AgCl) based on its LSV. The reaction current (at 0.4 V) was divided by the true surface area and actual mass of Pt to obtain the SA. The reaction current (at 0.4 V) was divided by the actual mass of Pt to obtain the MA [25,26]. The SA was calculated using Equation (1) as follows,

SA = IECSA × Pt loading amount(1)

while the MA was calculated using Equation (2) as follows:

MA = I × Pt loading amount(2)

Here, I is the reaction current at 0.4 V in the LSV curve (MA), and the Pt loading amount represents the amount of Pt in the electrode (mg).

The MA and SA values shown in Figure 4d were obtained by synchronizing the kinetic current with the mass of Pt and ECSA, respectively. The Pt/HC55 catalyst exhibited the best ORR performance, showing MA and SA values of 66.2 mA/mg_Pt_ and 8.15 mA/cm^2^, respectively. For comparison, the MA and SA values of Pt/CB, Pt/srGO, and Pt/CNTs were 23.2 mA/mg_Pt_ and 1.90 mA/cm^2^; 57.9 mA/mg_Pt_ and 6.95 mA/cm^2^; and 60.9 mA/mg_Pt_ and 9.72 mA/cm^2^, respectively. All characteristics of the different catalysts are listed in Table 2.

Catalysts Pt/HC73, which had a high Pt/srGO ratio, and Pt/HC37, which had a high Pt/CNT ratio, showed lower ORR activities than those of Pt/srGO, Pt/CNTs, and Pt/HC55. Thus, the synthesized catalysts exhibited higher ORR activities than Pt/CB. In addition, it was confirmed that Pt/HC55—in which CNTs, which exhibit high electrical conductivity, and srGO, which has a high specific surface area, were combined in equal amounts—showed increased ORR activity. In this catalyst, the three-dimensional CNTs were well dispersed over the two-dimensional srGO layers and acted as spacers, thus enhancing electron diffusion. As a result, its activity was higher than that of Pt/srGO and Pt/CNTs [27,28]. However, in the case of Pt/HC73, in which the ratio of srGO to CNTs was 7:3, the presence of an excessive amount of srGO resulted in CNT aggregation (Figure S1a), which reduced the performance of the catalyst. In the case of Pt/HC37, which had a srGO/CNT ratio of 3:7, the addition of too many CNTs resulted in the srGO sheets being completely covered by the CNTs, which increased the diffusion resistance of the electrons and hence lowered the activity (Figure S1b).

**Figure 5:**
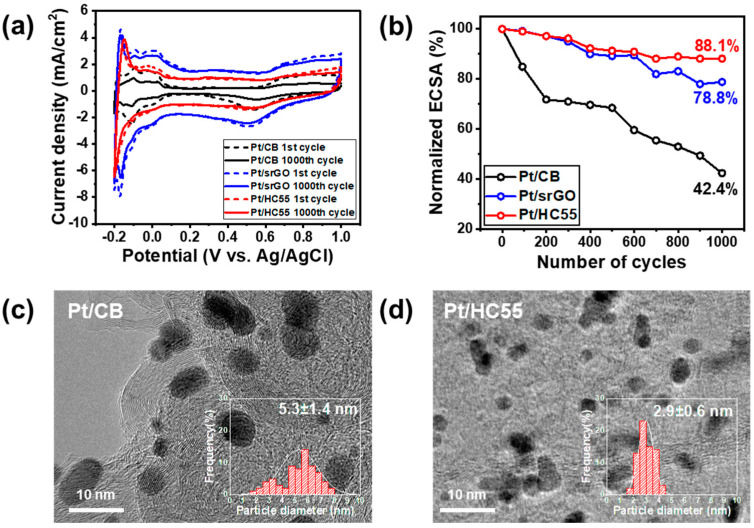
(**a**) CV curves of Pt/srGO, Pt/HC55, and Pt/CB before and after 1000 cycles of ADT in N_2_-saturated 0.5 M H_2_SO_4_ at scan rate of 50 mV/s. (**b**) Normalized ECSAs of catalysts measured over 1000 cycles of ADT. TEM images and Pt particle size distribution histograms of (**c**) Pt/CB and (**d**) Pt/HC55 after 1000 cycles of ADT.

The stability of Pt/CB, Pt/srGO, Pt/CNTs, and Pt/HC55 was investigated using the ADT, which was performed for 1000 cycles from −0.2 to 1.0 V (versus Ag/AgCl). The CV curves were measured in a N_2_-saturated 0.5 M H_2_SO_4_ solution at a scan rate of 50 V/s before and after 1000 ADT cycles, as shown in Figure 5a and Figure S4. The normalized ECSA values as determined after 1000 cycles are shown in Figure 5b. The ECSA of Pt/HC55 decreased by 11.9%, while those of Pt/srGO and Pt/CB decreased by 21.2% and 57.6%, respectively. TEM images of Pt/HC55 and Pt/CB were obtained before and after the ADT. The Pt nanoparticles in the commercial Pt/CB catalyst were agglomerated into large, irregular nanoparticles after the ADT, as shown in Figure 5c. However, the Pt nanoparticles of Pt/HC55 exhibited a smaller degree of aggregation than those of Pt/CB after the ADT, as shown in Figure 5d. The average Pt size of the Pt/CB catalyst was increased to 5.3 nm (150% increase) after ADT; however, that of the Pt/HC55 catalyst increased to 2.9 nm (26% increase), which clearly showed the higher durability of the Pt/HC55 catalyst. In the case of Pt/CB, amorphous carbon black has many sp3 bonds, and it is therefore more vulnerable to decomposition compared to the ordered sp2 carbon structure. In the ADT result of Pt/srGO, a large loss of 21.2% occurred due to the many defects in the sp2 bond produced during the synthesis process of rGO. Compared to rGO, CNTs have strong corrosion resistance because there are fewer defect sites on the surface, and the addition of CNTs in rGO further improved the durability and the electrical conductivity of the catalyst [29,30,31,32]. Thus, srGO, which has a high specific surface area, and the CNTs, which show a high degree of crystallinity, suppressed carbon oxidation and resulted in higher durability than that of the existing Pt/CB and Pt/srGO catalysts.

## 3. Materials and Methods

### 3.1. Catalyst Synthesis

The catalyst components were synthesized by the polyol method using microwaves (Multiwave 5000, Anton Paar GmbH, Graz, Austria). The amount of Pt in the catalysts was fixed at 20 wt%. First, 80 mg of srGO, CNTs, or CB was mixed with 40 mL of ethyl glycol under sonication for 1 h. A Pt precursor, namely H_2_PtCl_6_∙6H_2_O (Sigma-Aldrich, St. Louis, MO, USA), was added to the resulting solutions under magnetic stirring for approximately 30 min. The pH of the solutions was adjusted to 12 using KOH. Finally, the prepared solutions were heated in a microwave heating system. The synthesized catalysts were washed with ethanol and deionized water and dried in a vacuum oven at 50 °C overnight. Next, the HCs were synthesized by combining Pt/srGO and Pt/CNTs. Catalysts Pt/HC73, Pt/HC55, and Pt/HC37 were obtained by combining Pt/srGO and the Pt/CNTs in ratios of 7:3, 5:5, and 3:7, respectively.

### 3.2. Characterization 

The surface morphologies and elemental compositions of the catalysts were analyzed using field-emission SEM (FE-SEM; Hitachi, Tokyo, Japan, SU8020) and TEM (JEOL Ltd., Tokyo, Japan, JEM-2100F), respectively. Raman spectroscopy (WITec, Ulm, Germany, alpha300s) was performed to identify the changes in the defects in srGO; the wavelength of the incident light was 532 nm. The electrical characteristics of the catalysts were evaluated using a source measure unit (Tektronix, Beaverton, OR, USA, Keithley 2400) in a pressure cell (M&S Vacuum, Goyang-si, Korea) under a uniaxial pressure of 5 MPa at room temperature. The crystallinities of the catalysts were determined using X-ray diffraction (XRD) analysis (Rigaku, Tokyo, Japan, Ultima IV/Rigaku), which was performed using Cu-Kα (λ = 0.15406 nm) radiation in the 2θ range of 10–85° at a scan rate of 1°/min. The Pt (wt%) concentration of the catalysts was measured using thermogravimetric analysis (Mettler Toledo, Greifensee, Switzerland, TGA/DSC1), which was performed in an air flow. The samples were heated from 3 °C to 850 °C at 10 °C/min and held at 200 °C for 2 h.

### 3.3. Electrochemical Measurements

Electrochemical measurements were performed on the catalysts using a half-cell system with three electrodes (BioLogic, Seyssinet-Pariset, France, VSP 300). A Pt wire and Ag/AgCl electrode were used as the counter and reference electrodes, respectively. The catalyst to be tested was coated on the surface of glassy carbon (GC) (AFE5T050GC, 5.0 mm disk OD, Pine Research, Durham, NC, USA), which was used as the working electrode. First, approximately 5 mg of the catalyst whose electrochemical activity was to be measured was dispersed in a mixture containing isopropyl alcohol (1 mL) and a 5 wt% Nafion solution (60 μL), and the mixture was sonicated for 30 min until a homogenous suspension was obtained. Next, this solution (20 μL) was drop-cast onto the GC electrode using a micropipette and dried at room temperature (27 °C). 

CV was performed at a scan rate of 20 mV/s from −0.2 to 1.0 V in N_2_-saturated 0.5 M H_2_SO_4_. LSV measurements were performed at a scan rate of 5 mV/s and rotating disk electrode (RDE) speed of 1600 rpm in O_2_-saturated 0.5 M H_2_SO_4_. Accelerated durability tests (ADTs) were performed at a scan rate of 20 mV/s for 1000 cycles from −0.2 to 1.0 V (versus Ag/AgCl) in 0.5 M H_2_SO_4_. The electrochemical surface area (ECSA) of Pt in the catalysts was calculated using the expression ECSA = Q_H_/(C × m), where Q_H_ (μC) is the charge required for hydrogen desorption during CV, C is the electrical charge (210 μC/cm^2^) for the monolayered adsorption of hydrogen onto the surfaces of the Pt nanocrystals, and m is the amount of Pt loaded on the working electrode. For the ORR, the mass and specific activity were obtained by normalizing i_k_ (obtained from the Koutecky–Levich equation (1/i = 1/i_d_ + 1/i_k_) with respect to Pt and the ECSA, respectively [33,34,35].

## 4. Conclusions

We synthesized highly active and durable HCs composed of srGO and CNTs as ORR catalysts for PEMFCs. Pt/srGO and Pt/CNTs were prepared by loading Pt nanoparticles onto srGO and CNTs using a polyol process. Subsequently, HCs with different Pt/CNT and Pt/srGO ratios were prepared by mechanically mixing the two components. The synthesized HCs consisted of Pt/CNTs well dispersed on Pt/srGO, with catalyst HC55, which was prepared using a Pt/srGO-to-Pt/CNT ratio of 5:5 exhibiting excellent ORR performance and high stability over 1000 ADT cycles. In particular, the normalized ECSA values, as determined after 1000 cycles of the ADT, showed that the ECSA of Pt/HC55 decreased by 11.9%, while those of Pt/srGO and Pt/C decreased by 21.2% and 57.6%, respectively. A detailed analysis of the structural and electrochemical properties of the HCs suggested that the synergetic effects of the high specific surface area of srGO and the excellent electrical conductivity of the CNTs were responsible for the enhanced efficiency and durability of the HCs. We believe that our study should aid efforts to improve the performance of existing PEMFCs and other types of fuel cells.

## Figures and Tables

**Table 1:** Brunauer–Emmet–Teller (BET) analysis results for Pt/srGO, Pt/CNTs, Pt/HC73, Pt/HC55, and Pt/HC37.

Sample	S_BET_ (m^2^/g)	Pore Volume (cm^3^/g)	Pore Size (nm)
Pt/srGO	700.9	3.278	18.80
Pt/HC73	664.3	3.074	18.60
Pt/HC55	334.0	1.637	19.60
Pt/HC37	245.4	1.105	18.01
Pt/CNT	210.2	0.950	18.08

**Table 2:** E_onset_, E_half-wave_, MA, and SA values of Pt/CB, Pt/srGO, Pt/CNTs, and Pt/HC55.

Sample	E_onset_ (mV)	E_half-wave_ (mV)	MA (mA/mg_Pt_)	SA (mA/cm^2^)
Pt/CB	489.6	381.05	23.2	1.90
Pt/srGO	612.6	568.8	57.9	6.95
Pt/CNTs	610.3	533.0	60.9	9.72
Pt/HC55	614.3	580.2	66.2	8.15

## Data Availability

The data that support the findings of this study are available from the corresponding authors upon reasonable request.

## Supplementary Materials

The following supporting information can be downloaded at: https://www.mdpi.com/article/10.3390/ijms232113312/s1.
