# Supplementary material for: Hybrid Carbon Supports Composed of Small Reduced Graphene Oxide and Carbon Nanotubes for Durable Oxygen Reduction Catalysts in Proton Exchange Membrane Fuel Cells
Source: Int J Mol Sci. 2022 Nov 1;23(21):13312. doi: 10.3390/ijms232113312 (PMC9657248; doi:10.3390/ijms232113312)
Supplement: Supplementary file 1 [file ijms-23-13312-s001.zip › ijms-1997398-supplementary.pdf]

Supplementary Information

Hybrid Carbon Supports Composed of Small Reduced Graphene Oxide and Carbon Nanotubes for Durable Oxygen Reduction Catalysts in Proton Exchange Membrane Fuel Cells

Su-Jeong Bak[a,1], Mingyu Son[a,b,1], Jeehoon Shin[a], Sun-I Kim[a], Jung Woo Lee[b,], Duck Hyun Lee[a,*]*

[a]*Green Materials and Processes R&D Group, Korea Institute of Industrial Technology, Ulsan 44413, Republic of Korea*

[b]*Department of Materials Science & Engineering, Pusan National University, Busan 46241, Republic of Korea*

* Address correspondence to: dulee@kitech.re.kr (D. H. Lee), jungwoolee@pusan.ac.kr (J. W. Lee)

[1] These authors contributed equally to this work

SUPPLEMENTARY FIGURES

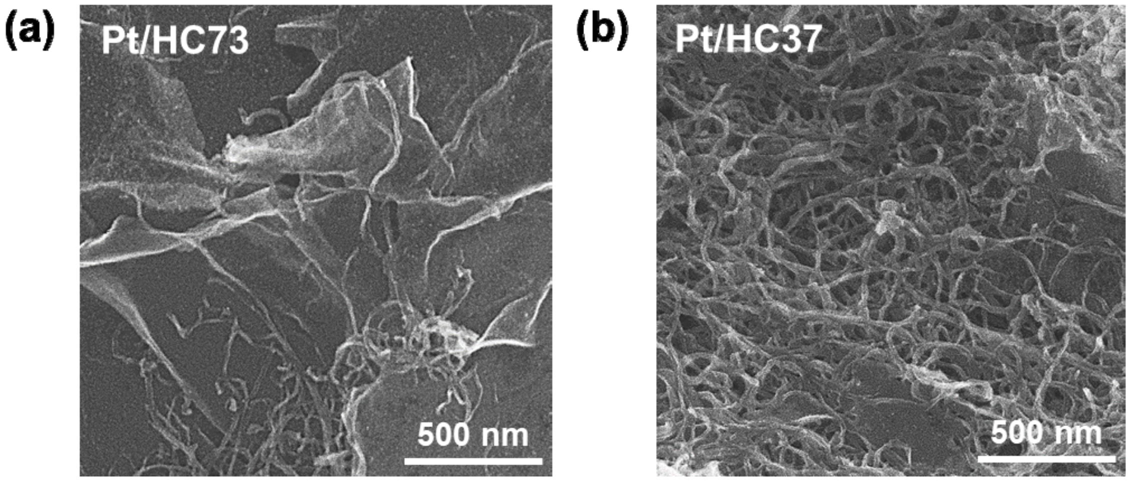

Figure S1. Scanning electron microscopy (SEM) images of the synthesized (a) Pt/HC73 and (b) Pt/HC37.

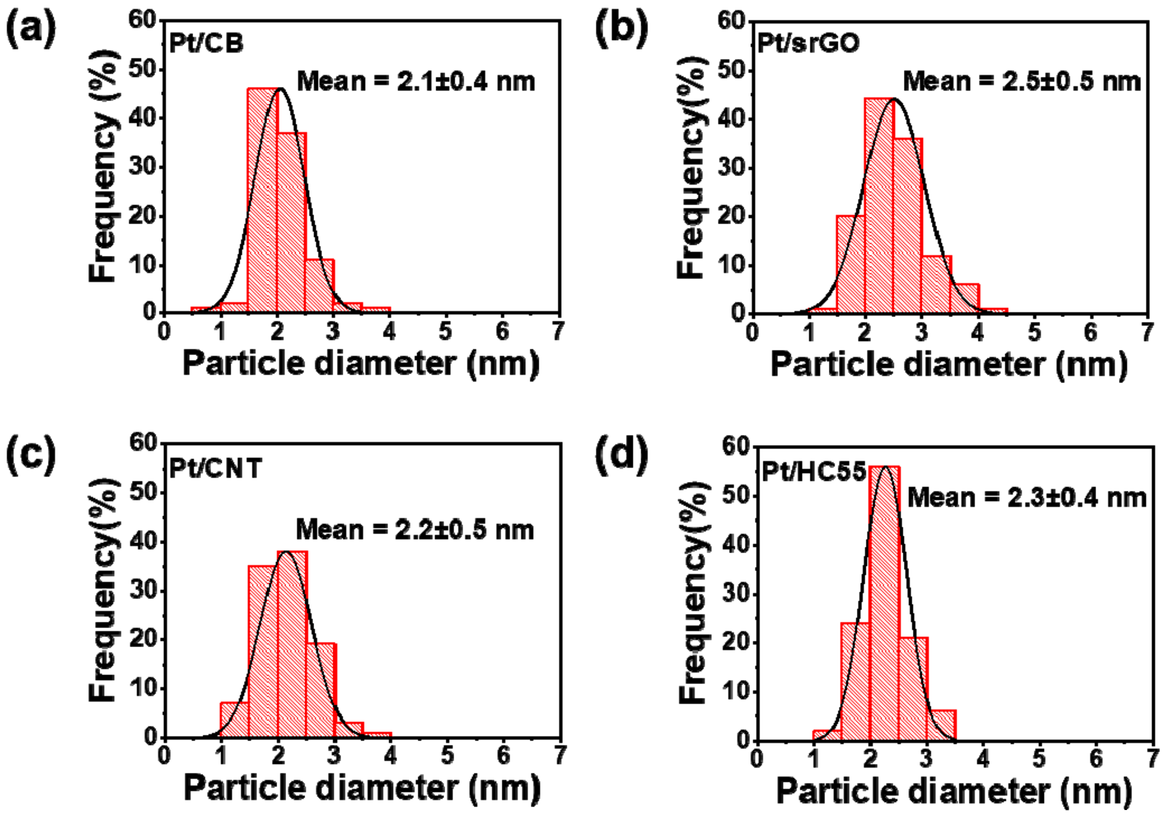

Figure S2. Pt particle size distribution histograms of (a) Pt/CB, (b) Pt/srGO, (c) Pt/CNT and (d) Pt/HC55.

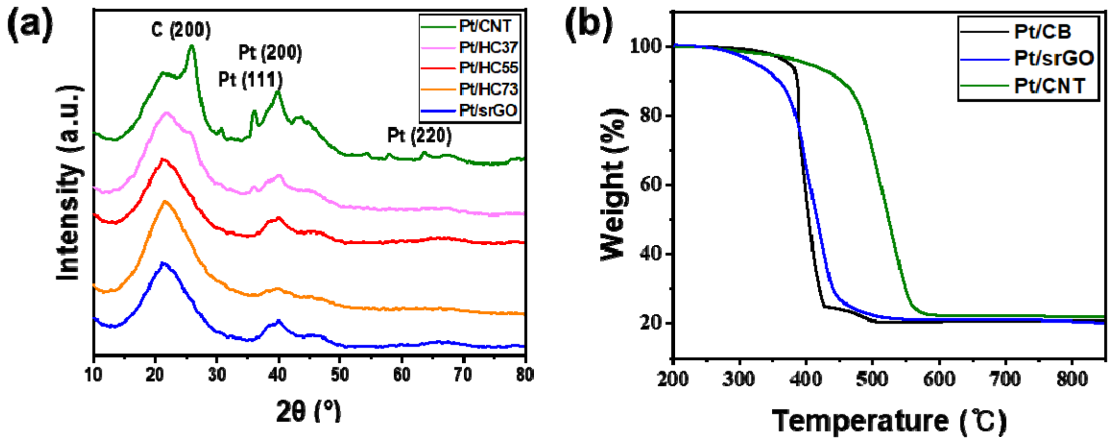

Figure S3. (a) X-ray diffraction patterns and (b) Thermogravimetric analysis (TGA) curves of th e synthesized catalysts.

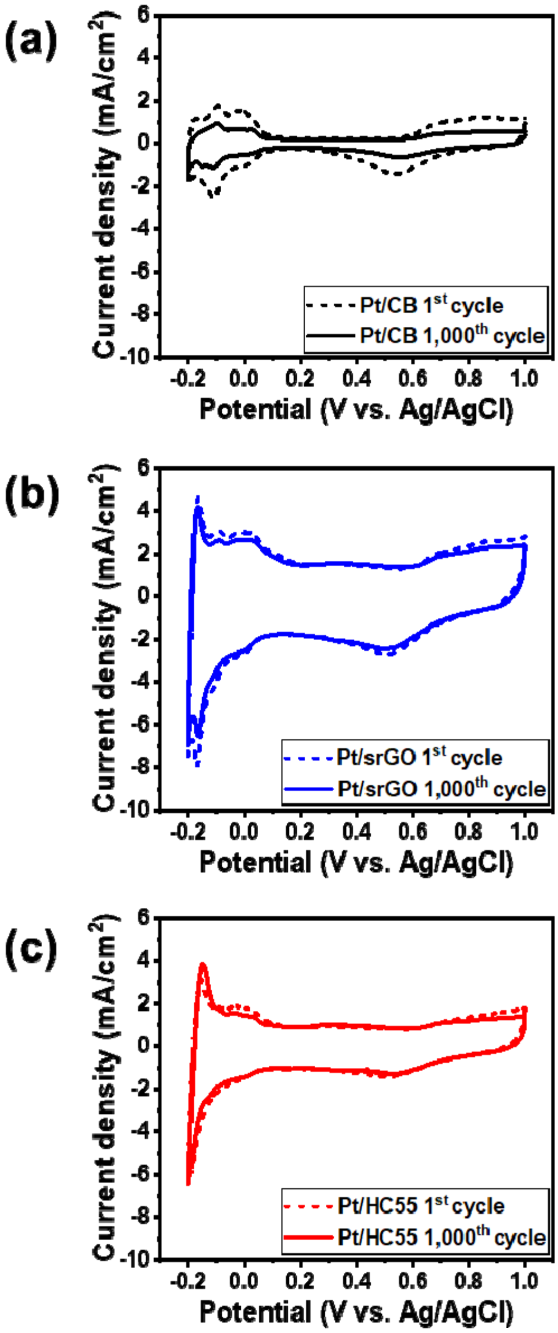

Figure S4. Cyclic voltammetry curves of (a) Pt/CB, (b) Pt/srGO, and (c) Pt/HC55 before and after the 1,000 cycles of accelerated durability tests in N_2-saturated 0.5 M H_2SO_4 at a scan rate of 50 mV/s.
